# Designing virtual patient based self-study quizzes covering learning goals in clinical diagnostic sciences for undergraduate medical students – the radiology example

**DOI:** 10.3205/zma001384

**Published:** 2020-12-03

**Authors:** Michaela Wagner-Menghin, Victor Szenes, Martina Scharitzer, Peter Pokieser

**Affiliations:** 1 Medical University Vienna, Teaching Center, Vienna, Austria; 2 Medical University Vienna, Department of Biomedical Imaging and Image-guided Therapy, Vienna, Austria; 3 Medical University Vienna, Radiologie Währing, Vienna, Austria; 4 Medical University Vienna, Institut für bildgebende Diagnostik, Sanatorium Hera, Vienna, Austria

**Keywords:** clinical diagnostic sciences, virtual patients, teaching radiology

## Abstract

**Background: **Diagnostic tests and examinations inform clinical decision making. Thus, an essential part of medical students’ workplace-based training is dedicated to core skills in clinical diagnostic sciences. Due to a reduction of clinical internships for fifth-year students in the wake of COVID-19 learning activities replacing this aspect of training were needed.

**Project description: **Virtual Patient online learning materials addressing clinical diagnostic sciences, specifically, radiology, were developed to prepare students for the transition to workplace-based learning. Three types of activities related to interprofessional patient treatment, showing how radiology knowledge improves the diagnosing and treatment of patients, were used to design the narrative of each virtual patient. The materials also showed students “how to learn” in the clinical workplace while showing “what to learn”. Students complete relevant tasks and compare their approach with experts’ approach in a self-directed way.

**Results:** Twenty self-study quizzes, accompanied by nine interactive Webinars were developed, providing 13% of the overall available replacement learning materials for the summer term 2020. In June 2020, 486 students completed the program and collected a mean share of 16% (SD=10) of their required credits by choosing to learn with these materials.

**Conclusion: **Developing virtual patients based on three types of clinical activities to prepare students for the transition to workplace based learning proved successful and allowed rapid development of learning materials. The presented online quiz format and webinar format showed high acceptance and interest among students.

## Zusammenfassung

**Hintergrund: **Ergebnisse aus diagnostischen Tests und Untersuchungen stellen relevante Informationen für die klinische Entscheidungsfindung dar. Daher ist ein wesentlicher Teil der arbeitsplatzbasierten Ausbildung von Medizinstudierenden dem Erwerb von Kernkompetenzen in klinischen Diagnosewissenschaften gewidmet. Da COVID-19 bedingt die klinischen Praktikumsplätze für Studierende im fünften Studienjahr reduziert wurden, war es notwendig, diese Teile der Ausbildung zu ersetzen.

**Projektbeschreibung: **Am Beispiel der Radiologie wurden online-Lernmaterialien zum Thema klinische Diagnosewissenschaften entwickelt, um Studierende durch die Arbeit mit virtuellen PatientInnen auf den Übergang zu arbeitsplatzbezogenem Lernen vorzubereiten. Zur Entwicklung der PatientInnenszenarien wurden drei für die interdisziplinäre PatientInnenbehandlung relevante Aktivitätstypen definiert. Die entwickelten Lernszenarien zeigen, was zu lernen ist und wie radiologisches Wissen die Diagnose und Behandlung von PatientInnen verbessert. Gleichzeitig zeigen die Szenarien den Studierenden, wie man am klinischen Arbeitsplatz lernt: Nach möglichst selbständiger Erledigung einer praxisrelevanten Aufgabe vergleichen Studierende die Übereinstimmung ihres Ansatzes mit einem von klinischen ExpertInnen vorgestellten Ansatz.

**Ergebnisse: **Die entwickelten zwanzig Selbstlernquizze und neun interaktiven Webinare entsprechen 13% der für Jahr 5 insgesamt angebotenen akademischen Ersatz-Stunden für das Sommersemester 2020. Im Juni 2020 schlossen 486 Studierende das Ersatzprogramm ab und sammelten einen durchschnittlichen Anteil von 16% (SD=10) ihrer benötigten akademischen Stunden durch Teilnahme an Quizzen und/oder Webinaren.

**Schlussfolgerung: **Der vorgestellte Ansatz, virtuelle PatientInnenszenarien zur Vorbereitung auf späteres arbeitsplatzbasiertes Lernen anhand prototypischer klinischer Aktivitätstypen zu entwickeln, kann als erfolgreich bezeichnet werden. Das aufgrund von Covid-19 sehr rasch entwickelte Online-Quiz-Format und das Webinar-Format zeigten hohe Akzeptanz und Interesse unter den Studierenden.

## 1. Introduction

During clinical decision making primary care physicians or medical specialists of various disciplines compile information from the patient’s history, the physical examination, and diagnostic procedures, which are performed and appraised by medical specialists. To make the best use of diagnostic procedures, all practising physicians should rely on skills related to clinical diagnostic sciences (CDS) as specified for undergraduate medical education [1], [2], [3].

### 1.1. Problem

Due to the outbreak of COVID-19 Austrian hospitals reduced the number of internships for fifth-year medical students, thus canceling their workplace-based learning of CDS. The Learning Development Team of the Teaching Center was approached – more than ten teams from different departments of the Medical University were approached – by the Director of the Curriculum to create replacement learning scenarios, covering some KLZÖ learning goals (Austrian National Learning Objectives Catalogue for Undergraduate Medical Education) with the following specifications:

1. *Online activities:* to comply with COVID-19 measures
2. *Self-directed learning:* to facilitate the transition to workplace-based learning

## 2. The project

The last author’s rich archive of radiological cases made it feasible to focus the development of virtual patient learning materials (VPs) on CDS related to radiology. 

### 2.1. Three types of clinical activity requiring CDS knowledge – the radiology example

The students’ motivation engaging in CDS learning goals varies greatly. Tasks like identifying anatomical structures and pathologies on radiological images might not seem relevant to some students. During class, teachers remediate such motivational difficulties by illustrating how CDS knowledge enhances clinical decisions. To foster students’ motivation in online learning, we structure the students’ tasks around three types of clinical activities, thus highlighting the specific CDS knowledge for decision making. 

#### 2.1.1. Selecting an imaging method pertinent to the clinical situation

All physicians referring to medical imaging need detailed knowledge about indications and difficulties in performing radiological investigations. 

##### 2.1.2. Identifying pathologies on images and communicating them

Radiologists carry out the radiological reporting, but all physicians need skills in recognizing abnormalities on radiological images.

##### 2.1.3. Supporting patients in the understanding of doctors’ or boards’ letters

All physicians need to talk about imaging with patients in a comprehensible manner (examples see table 1 (Tab. 1)).

#### 2.2. Simulating self-directed learning in the workplace with self-study quizzes 

During workplace-based learning students observe the decision making of senior doctors. Students participate in this process in a self-directed way by attempting problem solving and by reflecting on work practices between together with the supervisor, creating knowledge as a shared activity. Since the transition to workplace-based learning is difficult for undergraduates [4], we propose VPs to show them “how to learn” in a self-directed way while showing them “what to learn”. While students complete relevant tasks with authentic patient information [5], [6], they compare their approach with the approach of experts using four categories ranging from 0% (no concordance, different approach) through 40% or 80% to 100% (similar approach) to self-score the quiz. Including an appropriate basic science question together with a clinical application question, activates the students’ basic sciences knowledge. A short teaching summary promotes learning. Self-Study Quizzes were presented using Moodle, or in interactive webinars (without concordance rating). 

#### 2.3. Evaluation 

To evaluate we prompt students to comment on each quiz freely; we also compare the mean share of credits earned by students choosing to participate in our learning activities with the share of credits provided by our learning materials within the overall replacement program. 

## 3. Results

### 3.1. Learning materials

Twenty self-study quizzes and nine interactive live webinars were developed, covering 16 KLZÖ-objectives (up to five objectives per VP). Three learning activity types highlighted the learner’s perspective during the development of the quizzes’ content. 

#### 3.2. Student participation 

By participating in all available replacement activities, one could overfulfill the requirements by about 75 percentage points. As our learning activities accounted for 13% of available credits, nobody was obliged to participate since there were alternatives available. In June 2020, 486 fifth-year students completed the replacement program and earned a mean share of credits by participating in our quizzes and webinars of 16% (standard deviation±10). Students chose our activities as compared to the alternatives with a similar preference. Students’ self-scoring resulted in a mean concordance score of 81% (SD=10%). On average 10% (SD=3%) of students commented per quiz, expressing predominately satisfaction (e.g. *great case, ☺ *) or neutrality (e.g. *“thanks”, “nothing in particular”*). 

## 4. Discussion

Defining three types of clinical activities helped in guiding the development of VPs requiring CDS skills related to radiology, which have previously been proposed for medical undergraduates [7]. The VP-based quizzes and webinars showed high acceptance among the students and can be improved based on the provided comments.

## 5. Conclusion

Our approach in guiding the development of online VPs to improve the transition of students to workplace-based learning demonstrates high acceptance among students. As such, the second edition for all quizzes is currently under preparation. Besides working on covering more of the KLZÖ learning goals, development has to focus on systematically describing how CDS knowledge is needed in varying clinical settings to systematically prepare students for the challenges they face related to learning in the clinical workplace.

## Competing interests

The authors declare that they have no competing interests. 

## Figures and Tables

**Table 1:**
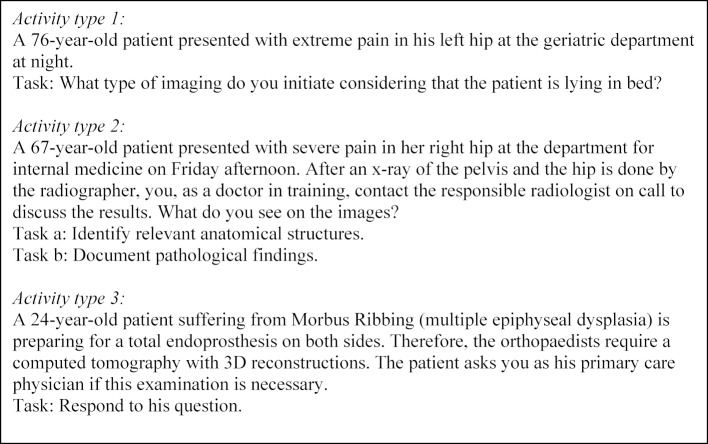
Activity typs

## References

[1] Medizinische Universität Graz, Medizinische Universität Wien, Medizinische Universität Innsbruck, Medizinische Fakultät Linz (2020). Klinischer Lernzielkatalog Österreichs.

[2] Wissing F (2018). Nationaler Kompetenzbasierter Lernzielkatalog Medizin und Zahnmedizin (NKLM/NKLZ). Bundesgesundheitsbl Gesundheitsforsch Gesundheitsschutz.

[3] Bürgi H, Rindlisbacher B, Bader C, Bloch R, Bosman F, Gasser C, Gerke W, Jumair JP, Im Hof V, Kaiser H, Lefebvre D, Schläppi P, Sottas B, Spinas GA, Stuck AE (2008). Swiss Catalogue of Learning Objectives for Undergraduate Medical Training.

[4] Teunissen PW, Westerman M (2011). Opportunity or threat: the ambiguity of the consequences of transitions in medical education. Med Educ.

[5] Hege I, Kononowicz AA, Tolks D, Edelbring S, Kuehlmeyer K (2016). A qualitative analysis of virtual patient descriptions in healthcare education based on a systematic literature review. BMC Med Educ.

[6] Urresti-Gundlach M, Tolks D, Kiessling C, Wagner-Menghin M, Härtl A, Hege I (2017). Do virtual patients prepare medical students for the real world? Development and application of a framework to compare a virtual patient collection with population data. BMC Med Educ.

[7] Alexander AG, Deas D, Lyons PE (2018). An Internet-Based Radiology Course in Medical School: Comparison of Academic Performance of Students on Campus Versus Those With Absenteeism Due to Residency Interviews. JMIR Med Educ.

